# A consistent pattern of slide effects in Illumina DNA methylation BeadChip array data

**DOI:** 10.1080/15592294.2023.2257437

**Published:** 2023-09-20

**Authors:** Julian Hecker, Sanghun Lee, Priyadarshini Kachroo, Dmitry Prokopenko, Anna Maaser-Hecker, Sharon M. Lutz, Georg Hahn, Rafael Irizarry, Scott T. Weiss, Dawn L. DeMeo, Christoph Lange

**Affiliations:** a Channing Division of Network Medicine, Brigham and Women’s Hospital and Harvard Medical School, Boston, MA, USA; b Department of Biostatistics, Harvard T.H. Chan School of Public Health, Boston, MA, USA; c Department of Medical Consilience, Division of Medicine, Graduate School, Dankook University, Yongin-si, South Korea; d Genetics and Aging Unit and McCance Center for Brain Health, Department of Neurology, Massachusetts General Hospital and Harvard Medical School, Boston, MA, USA; e Department of Population Medicine, PRecisiOn Medicine Translational Research (PROMoTeR) Center, Harvard Pilgrim Health Care and Harvard Medical School, Boston, MA, USA; f Department of Data Sciences, Dana-Farber Cancer Institute, Boston, MA, USA

**Keywords:** DNA methylation, methylation arrays, batch effects, slide effects, EWAS

## Abstract

**Background:** Recent studies have identified thousands of associations between DNA methylation CpGs and complex diseases/traits, emphasizing the critical role of epigenetics in understanding disease aetiology and identifying biomarkers. However, association analyses based on methylation array data are susceptible to batch/slide effects, which can lead to inflated false positive rates or reduced statistical power

**Results:** We use multiple DNA methylation datasets based on the popular Illumina Infinium MethylationEPIC BeadChip array to describe consistent patterns and the joint distribution of slide effects across CpGs, confirming and extending previous results. The susceptible CpGs overlap with the Illumina Infinium HumanMethylation450 BeadChip array content.

**Conclusions:** Our findings reveal systematic patterns in slide effects. The observations provide further insights into the characteristics of these effects and can improve existing adjustment approaches.

## Introduction

Epigenome-wide association studies (EWAS) are an increasingly popular approach to characterizing the association between DNA methylation and complex diseases and traits [1]. DNA methylation data are routinely generated by methylation arrays such as the Illumina Infinium HumanMethylation450 BeadChip and the Infinium MethylationEPIC BeadChip array. When using methylation array data, one of the challenges of EWAS is to control for batch effects. Batch effects describe systematic differences between the measurements of different batches of experimental data due to technical factors [2]. Batch effects introduce increased within-group variances and can lead to reduced statistical power in the analysis of the experiment. Moreover, if group comparisons as part of the experimental design are aligned with these batches, false positive association findings can occur.

Batch effects can arise due to several sources of variability, including human factors, laboratory protocols, environmental factors, and the technologies used. Another challenge in this context is to define the appropriate batch level. For example, the batch structure can be introduced at the plate, slide, or positional level. Recently, Ross et al. considered the batch structure for DNA methylation data at the slide level and performed comprehensive analyses to characterize slide effects using DNA methylation data generated by both arrays [2]. Here, we use multiple DNA methylation datasets to confirm and extend these results, including a characterization of the joint distribution of slide effects and the corresponding consistency across datasets. Our findings emphasize the value of understanding and exploiting the structure of slide effects in methylation array data to enable valid analyses.

## Materials and methods

Our analyses aim to investigate slide effects in DNA methylation data generated by popular methylation arrays and to analyse the consistency and characteristics of these effects.

### Study populations

We incorporated cord blood-based DNA methylation for the Vitamin D Antenatal Asthma Reduction Trial (VDAART) [3], whole blood-based DNA methylation for the Genetic Epidemiology of Asthma in Costa Rica Study (GACRS) [4] and the Childhood Asthma Management Program (CAMP) [5], as well as cord blood mononuclear cells (CBMC) and peripheral blood mononuclear cells (PBMC) based (collected at age 7) methylation data for the Urban Environment and Childhood Asthma (URECA) birth cohort study [6] (Gene Expression Omnibus GSE132181). GACRS and CAMP consist of family trio data; we split these cohorts into offspring, mothers, and fathers. The analyses were performed for these sub-cohorts individually. Further details regarding the cohorts are provided in the Supplementary Methods.

### DNA methylation data

For all cohorts, methylation data were generated using the Illumina Infinium MethylationEPIC BeadChip array (Illumina, San Diego, CA). Methylation data were processed, normalized, and quality-controlled using slightly different procedures (see Supplementary Methods). This variation of processing procedures provides additional robustness to our findings regarding the consistency of the effects across datasets and quality control protocols.

### Slide effect analysis

We analysed the impact of slide effects on methylation measurements in all cohorts and compared the results across datasets. The analyses were based on linear mixed models where rank inverse normal transformed methylation values described the outcome, and slide effects are modelled as random effects. We rank inverse normal transformed data to address the assumption of normally distributed data for linear mixed models. Before the transformation, the beta-values were transformed to the M-value scale [7] and adjusted for age (if applicable), sex (if applicable), ethnicity/race (if applicable), and estimated cell-type proportions using linear regression (see Supplementary Methods, Adjustment Methods I).

Based on the results for the linear mixed model in each (sub-)cohort, we created the following non-overlapping sets of CpGs: $S_{0-20},S_{20-40},S_{40-60},S_{60-80,}$ and $S_{80-100}$. Here, $S_{80-100}$ contains all CpGs where at least one cohort analysis indicated more or equal to 80% of variance explained by slide effects. The set $S_{60-80,}$ is defined accordingly with a lower threshold of 60% and excluding all CpGs in $S_{80-100}$. The definitions of the sets $S_{0-20}$, $S_{20-40}$, and $S_{40-60}$ follow the same approach. Furthermore, we defined the set $S_{\mathrm{high}}$ of all CpGs for which at least 7 out of 9 datasets showed at least 60% explained variance by slide. This set $S_{\mathrm{high}}$ thus represents CpGs with highly consistent and strong slide effect susceptibility.

Furthermore, we repeated all analyses based on data that were corrected for slide effects using ComBat [8], and data where the first ten methylome-wide principal components from a principal component analysis (PCA) were included in the regression adjustment (Supplementary Methods, Adjustment Methods II and III, respectively). These two additional analyses investigate the sufficiency of these slide effect adjustments, especially for the CpGs that are strongly impacted by the slide structure.

### Incorporation of external data and downstream analyses

We also incorporated the results by Ross et al. to analyse the relationship between our results and their findings [2]. Moreover, we described the identified slide effect susceptible CpGs with respect to genomic locations and characteristics, reported EWAS associations, and their joint distribution.

### Statistical tests

We performed several enrichment analyses based on the inferred non-overlapping sets $S_{0-20},S_{20-40},S_{40-60},S_{60-80,}$ and $S_{80-100}$. These tests were based on chi-squared tests in the case of counts/proportions and ANOVA for quantitative measurements.

## Results

After quality control, we kept 492 VDAART samples, 195 URECA PBMC samples, 194 URECA CBMC samples, 788 GACRS offspring, 174 GACRS mothers, 167 GACRS fathers, 720 CAMP offspring, 367 CAMP mothers, and 381 CAMP fathers for our analyses.

### Consistent slide effects across studies

**Figure 1.**
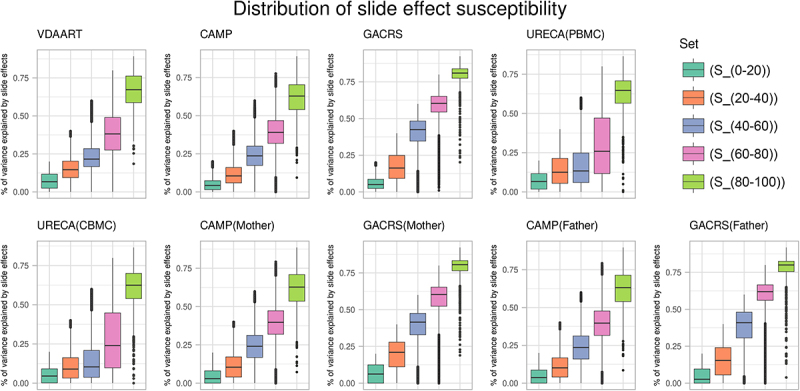
Proportions of variance explained by slide effects in VDAART, CAMP (offspring, mothers, fathers), GACRS (offspring, mothers, fathers), and URECA (PBMC and CBMC), partitioned by $S_{0-20},S_{20-40},S_{40-60},S_{60-80,}$ and $S_{80-100}$.

The number of CpGs in the sets $S_{0-20},S_{20-40},S_{60-80,}$ and $S_{80-100}$ were 250,972, 370,770, 160,713, 33,667, and 1,685, respectively (results based on Adjustment Method I, see Methods). The set $S_{\mathrm{high}}$ contained 1,578 CpGs. In Figure 1, we plotted the variance explained by slide effects in VDAART, GACRS (offspring, mothers, fathers), CAMP (offspring, mothers, fathers), and URECA (CBMC and PBMC) for all CpGs, stratified by the sets $S_{0-20},S_{20-40},S_{40-60},S_{60-80,}$ and $S_{80-100}$. The plots demonstrate the strong consistency of slide effects across the different datasets, reflected by the observation that the estimated slide effect magnitudes are remarkably similar across these sets. Moreover, we plotted all pairwise comparisons of estimated proportions of variance explained by slide effects between the nine sub-cohorts in Supplementary Figures S1–4.

As described in the Methods section, the DNA methylation measurements were adjusted for the variables age, sex, race, and cell type proportion estimates (each, if applicable) before the analysis. To perform additional robustness checks, we investigated the association between the first two principal components of CpGs in $S_{\mathrm{high}}$ and each of listed variables in VDAART, GACRS offspring, and CAMP offspring data. The first two principal components of CpGs in $S_{\mathrm{high}}$ capture the identified and highly correlated slide effects, but these variables did not significantly correlate with the variables. This provides further evidence that insufficient adjustments for age, sex, race, or cell type proportion estimates do not drive the results. In addition, we investigated the overlap between our inferred sets of CpGs and the list of probes included in established and popular epigenetic clocks (*Horvath* [9], *Hannum* [10], *and EpiTOC* [11] in the *cgageR* R package). There was no overlap between $S_{\mathrm{high}}$ and the *Hannum* and *EpiTOC* clocks, and only one CpG from the *Horvath* clock in $S_{\mathrm{high}}$, confirming no significant enrichment.

### Slide effect results consistent with previous analyses

Ross et al. performed closely related analyses and provided reference data that reports the amount of variance attributable to slide effects, as well as absolute mean difference values before and after slide effect adjustments, for five different DNA methylation datasets (based on the Illumina Infinium HumanMethylation 450 and MethylationEPIC BeadChip array). Their analysis also included URECA data. In Supplementary Figures S5–9, we compared our results with their findings by plotting the described quantities according to the inferred sets $S_{0-20},S_{20-40},S_{40-60},S_{60-80,}$ and $S_{80-100}$. These plots show that the results are in line with our observations, with strong replication in BodyFatness (EPIC) and EpiSCOPE (450), as well as their URECA results. These analyses reduce the possibility that the patterns are solely driven as a byproduct of the normalization procedure or study specifics and provide more evidence that the structure of slide effects is consistent between datasets. Since EpiSCOPE data were generated using the Methylation450 BeadChip array, it also shows consistency across both arrays.

Furthermore, our results are consistent with analyses performed by Higgins-Chen et al. in the context of epigenetic age prediction [12]. Higgins-Chen et al. analysed interclass correlation coefficients (ICCs) based on technical replicates to improve epigenetic clock constructions. For this analysis, they filtered a set of 78,464 CpGs shared between the Illumina MethylationEPIC and HumanMethylation450 BeadChip arrays and provided the ICCs for these probes in their supplementary material. In Supplementary Figure S10, we plotted the densities of ICCs for each set of CpGs in our analysis, also including ICCs from other studies as shared by Higgins-Chen et al. [13–15]. The plots demonstrate that increasing variance explained by slide effects in our investigation corresponds to decreasing ICCs in these independent analysis datasets, providing additional evidence for our findings. Formal testing using ANOVA demonstrates highly significant differences between our non-overlapping sets of CpGs with respect to the ICCs from the Higgins-Chen et al. main analysis (*p* < 2.2e-16).

Illumina recently introduced the Infinium MethylationEPIC v2.0 kit, and this update, besides other improvements, resulted in the exclusion of specific probes. We downloaded the list of removed probes from the Illumina support website (see data availability statement) and investigated the overlap with our sets of CpGs. Interestingly, CpGs in $S_{60-80}$ and $S_{80-100}$ were more likely to be excluded compared to $S_{0-20}$ and $S_{20-40}$ (21% and 44% compared to 13% and 11%). The CpGs were selected for removal based on the analyses performed by Zhou, Laird, and Shen [16]. We downloaded their corresponding supplementary material and observed that they reported a high enrichment for probes for which the 30bp 3’-subsequence of the respective probe is non-unique (23.6% of CpGs in $S_{80-100}$ compared to 0.7% in $S_{0-20}$, *p* < 2.2e-16).

### Joint distribution of slide effects reveals significant pattern

Besides the marginal impact of slide effects on individual CpGs, we also investigated the joint distribution of slide effects across the CpGs in the set $S_{80-100}$ (listed in Supplementary Table S1). We observed that the CpGs in this set showed strong between-CpG correlation in all datasets. This high correlation suggests that the influence of slides is consistent between these CpGs, introducing spurious co-methylation patterns. To investigate this in more detail, we extracted the fitted random effects from the mixed model-based approach. Next, we performed principal component analysis (PCA) of the estimated slide effects (fitted random effects). The first two principal components explained between 76.7% and 92.2% of the variance of estimated slide effects in the nine sub-cohorts.

**Figure 2.**
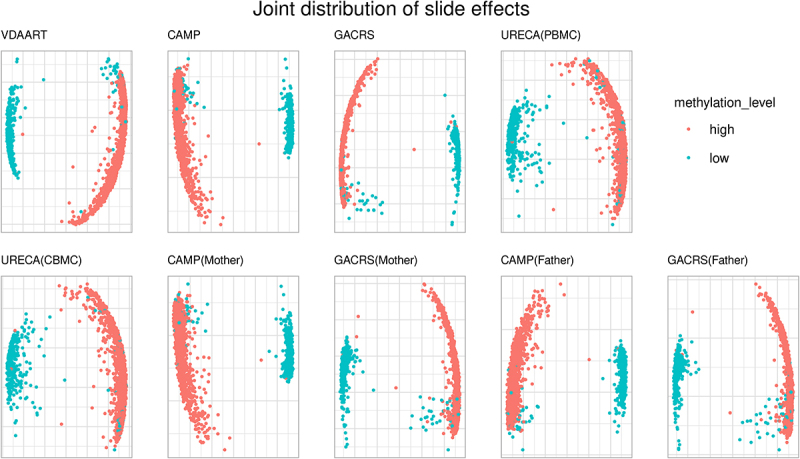
PCA plot (first two PCs) of estimated slide effects (mixed model random effects) in $S_{80\_100}$, colored by median methylation beta-values (50% cutoff) in the respective cohort.

The plot of the first two principal components is displayed in Figure 2. We coloured the points according to the overall median methylation beta-value level in the respective cohort: median below 50% (low) and above 50% (high). The results demonstrate that the slide effects are not independent between CpGs and reveal a significant pattern depending on the overall methylation level. The corresponding co-methylation/correlation coefficients between the CpGs in $S_{80-100}$ are summarized in Supplementary Figure S11, consistent with the previous observation.

In summary, although slide and batch effects have been described several times in the literature, our findings represent novel insights into these effects. The novelty of our findings is that the effect of slides is aligned across a large number of CpGs on different chromosomes, depending on the overall methylation level. This introduces spurious correlation (co-methylation) between these slide effect susceptible CpGs. While this observation is of independent interest, it also provides the basis for improved adjustment approaches. We further investigated and illustrated this in the simulation study.

### Characteristics of the slide susceptible CpGs

For further downstream analyses, we focused on the set $S_{\mathrm{high}}$ (Supplementary Table S2). The CpGs in this set are distributed across all chromosomes (including the X-chromosome). We analysed enrichments of genomic locations using the EWAS Toolkit [17]; the results are described in Supplementary Table S3. The CpGs are highly enriched for CpG Islands, 1^st^ Exon, and transcription start site (TSS) locations. Consistently, using additional annotation data provided by Illumina, we observed that Infinium type I probes are highly overrepresented in $S_{\mathrm{high}}$ compared to $S_{0-20}$ (53% vs. 8%, *p* < 2.2e-16). The same is true for Promoter-associated CpGs (29% vs. 7%, *p* < 2.2e-16) and red colour channel (43% vs. 5%, *p* < 2.2e-16). These observed characteristics are in line with the investigations by Ross et al. [2]. Their analysis also showed that, besides Infinium design (Type I or II), three other factors significantly associate with slide-effect-susceptibility: cross-hybridization due to highly homologous sequences, number of CpG sites internal to the probe, and probe melting temperature.

Additionally, we investigated the relationship between CpG-specific means and variation and the impact of slide effects. For this investigation, we computed the mean and corrected standard deviations for all CpGs in VDAART, GACRS offspring, and CAMP offspring data. The mean beta-values were computed on the beta-scale after adjustment for confounding variables (Adjustment Method I). The corrected standard deviation was computed as the corresponding estimated standard deviation but corrected for the remaining fraction of variation after slide effect adjustment (mixed model results). The corresponding density plots stratified by the sets of CpGs are visualized in Supplementary Figure S12. The results show that CpGs in $S_{80\_100}$ tend to be higher methylated and have increased variation. The plots are consistent across the three cohorts.

Finally, considering all CpGs in a radius of 100 basepairs around a CpG in $S_{\mathrm{high}}$, we notice a highly significant enrichment of larger slide effects compared to the baseline distribution (*p* < 2.2e-16), in line with the common observation of local co-methylation patterns in DNA methylation data.

### Overlap with EWAS results

Moreover, we analysed the CpGs in $S_{\mathrm{high}}$ for an overrepresentation of EWAS results [17]; Supplementary Table S4 reports the results of this analysis. The reported CpGs significantly overlap with several EWAS findings, covering both disease and exposure-related traits. In addition, we selected exemplary epigenetic association studies in which reported CpGs overlap with $S_{\mathrm{high}}($EWAS atlas association file download on 1 January 2023). For example, the EWAS atlas association database lists 118 CpGs identified by Novakovic et al. to be associated with assisted reproductive technologies [18]. Notably, 25 out of 118 CpGs are contained in $S_{\mathrm{high}}$. Furthermore, McCartney et al. performed an EWAS of sex-specific chronological ageing and published several association results for age, sex, and age-by-sex interaction outcomes [19]. Using the published summary statistics for the sex-specific analysis (sex as the outcome) [19], we extracted summary statistics for 1,270 CpGs in $S_{\mathrm{high}}$. Out of these 1,270 CpGs, 109 had a significant sex-association p-value *p* < 1e-08 in the discovery analysis, the corresponding number in the replication analysis was 643. For the replication analysis, this represents a strong enrichment of reported associations in $S_{\mathrm{high}}$ compared to the remaining methylome-wide results (*p* < 2.2e-16). These observations emphasize that the identified slide effect susceptible CpGs are of biological interest and have been reported in the EWAS literature. We note that the corresponding analyses addressed batch/slide effects in their analyses, but careful interpretation and replication are needed in future studies.

Ross et al. discussed the overlap between their identified slide effect susceptible CpGs and EWAS results using PubMed queries. They explicitly picked cg11963436, cg18368637, and cg22385669, which are in the sets $S_{60-80}$, $S_{80-100}$, and $S_{40-60}$, confirming the slide susceptibility of these CpGs based on our analysis.

### Standard adjustment methods can be insufficient

Repeating the mixed model-based analyses using data that were adjusted via ComBat or the top 10 methylome-wide principal components (Adjustment Methods II and III, Supplementary Methods), we observe that both approaches using the standard implementation might not be sufficient in practice to correct for the strong slide effects in $S_{80-100}$ (Supplementary Figures S13 and S14) and guard the analyses against spurious findings.

### Simulation study illustrates the utilization of slide-effect-susceptible CpGs for improved adjustment

We performed a simulation study to illustrate the utilization of our findings for improving slide effect adjustments. The simulations were based on the VDAART methylation data adjusted for sex, cell type proportions, and race (Adjustment Method I). We divided the CpGs of all autosomal chromosomes into two groups: one comprising chromosomes with odd numbers ($C_{\mathrm{odd}})$ and one with chromosomes with even numbers ($C_{\mathrm{even}}$). Next, we extracted the fitted random slide effects from the mixed model analysis (see above) for all CpGs on $C_{\mathrm{even}}$. These highly correlated effects were reduced to the first corresponding principal component. The phenotype was then simulated based on a standard normal variable plus this first principal component of effects (using the slide mapping in VDAART data). Therefore, the simulated phenotype is driven by approximated slide effects and random noise.

We performed association analysis using the methylation values for CpGs on $C_{\mathrm{odd}}$ and the simulated phenotype. The association analyses incorporated the following five different approaches; the corresponding adjustment methods used CpG data for $C_{\mathrm{odd}}$ only: plain (no correction, linear regression), PC_10 (first ten principal components based on all CpGs on $C_{\mathrm{odd}}$ as covariates in linear regression), PC_high (first principal component based on CpGs on $C_{\mathrm{odd}}$ in $S_{\mathrm{high}}$ as a covariate in linear regression), ComBat (ComBat adjustment based on CpGs on $C_{\mathrm{odd}}$ on M-value scale, linear regression), and ComBat_high (ComBat adjustment based on all CpGs on $C_{\mathrm{odd}}$ performed in $S_{\mathrm{high}}$ and all other CpGs separately on M-value scale, linear regression). We randomly selected (with replacement) 10,000 CpGs from each set by $S_{0-20},S_{20-40}$ and $S_{80-100}$ on $C_{\mathrm{odd}}$ and tested the CpGs for association with the phenotype. The phenotype was freshly drawn for each test separately. In Supplementary Figure S15, we plotted the quantile-quantile-plots for each set of CpGs based on the results for the five association test approaches. The plots show that the ‘plain’ approach without slide-effect-adjustment is highly inflated. PC_10 is able to control the type 1 error in the first three sets $S_{0-20},S_{20-40},$ and $S_{40-60}$, but shows inflation in $S_{60-80}$ and $S_{80-100}$. PC_high provides valid results across all sets. ComBat and ComBat_high show similar results across the first three sets $S_{0-20},S_{20-40},$ and $S_{40-60}$, but are inflated in $S_{60-80}$ and $S_{80-100}$. ComBat_high performs better in these two sets, since the adjustment was performed in all CpGs in $S_{\mathrm{high}}$ and all other CpGs separately (for CpGs in S_high, ComBat_high provided valid type 1 error rates). We also repeated the simulations based on methylation values on the M-value scale, but the results were qualitatively the same (Supplementary Figure S16).

Overall, this simulation shows that the systematic pattern of slide effects enables extracting the low-dimensional effect structure based on a small set of highly influenced CpGs. This low-dimensional information can be used as (additional) covariates to adjust EWAS. In our simulated example, a single covariate was sufficient to provide valid results. We emphasize again that the association analysis and adjustment were using data for CpGs on $C_{\mathrm{odd}}$ only, whereas the phenotype was simulated based on extracted slide effects on CpGs on $C_{\mathrm{even}}.$

## Discussion

We analysed the impact and consistency of slide effects on DNA methylation CpGs across multiple datasets based on Illumina Infinium MethylationEPIC BeadChip array data. Our analyses reveal a consistent set of CpGs that are highly susceptible to slide effects. Moreover, the joint distribution shows that the slide effects on these CpGs follow a specific pattern that introduces a spurious co-methylation structure, depending on the overall methylation status of the CpGs. The CpGs are enriched for Promoter-associated and Infinium type I CpGs. Our findings are in line with the results by Ross et al. that cover the MethylationEPIC as well as the HumanMethylation450 BeadChip array [2].

However, the investigations in this work have the following limitations. First, the datasets included in our analyses are solely blood-based, and the consideration of other tissues is missing in our investigations. Second, GACRS, CAMP, and VDAART data were processed similarly, while the identification of persistent slide effects requires the consideration of a maximum variety of processing and normalization techniques. Moreover, our slide effect analyses ignore the batch structure at the plate and slide position level and focus on slide as the defining structure only. We refer to the extensive and comprehensive investigations by Ross et al. for further insights into these other batch-level effects and a more detailed discussion of the factors associated with batch/slide effects [2].

Despite these limitations, our results are supported by the consistency across the studies in our analysis, the external results by Ross et al. [2], and downstream analyses that demonstrate the plausibility of the findings. Since the identified CpGs also overlap with previously reported EWAS associations, we re-emphasize that careful study design and adjustment methods are needed to account for these technical factors. Importantly, combining our novel insights with established adjustment/correction methods, such as ComBat [8] or PCA, has the potential to improve the robustness of future EWAS. We illustrated this approach in our simulation study where we constructed adjustment covariates. The sets $S_{80-100}$/$S_{\mathrm{high}}$ and a procedure to construct these covariates are available in the R package *SeffCovar* (https://github.com/julianhecker/SeffCovar).

## Supplementary Material

Supplemental Material

## Data Availability

GACRS and CAMP: All TOPMed data can be requested for access and can be made available through the TOPMed consortium after careful review and approval by the TOPMed Data Access Committee (https://topmed.nhlbi.nih.gov/). Participant consent and Data Use Limitations differ within and across TOPMed studies and should be requested individually. Additional documentation, such as local IRB approval and/or letters of collaboration with the primary study PI(s) may be required. DNA methylation data for the URECA cohort is available in the Gene Expression Omnibus (GEO) repository under the accession number GSE132181 (https://www.ncbi.nlm.nih.gov/geo/query/acc.cgi?acc=GSE132181). EWAS atlas: https://ngdc.cncb.ac.cn/ewas/atlas. EWAS Toolkit: https://ngdc.cncb.ac.cn/ewas/toolkit. Illumina EPIC v1.0 support files: https://support.illumina.com/downloads/infinium-methylationepic-v1-0-product-files.html. Illumina EPIC v2.0 support files: https://emea.support.illumina.com/downloads/infinium-methylationepic-v2-0-product-files.html. R statistical software, version 4.1.0: https://www.r-project.org/. *cgageR* R package: https://rdrr.io/github/metamaden/cgageR/.
