# Supplementary material for: A consistent pattern of slide effects in Illumina DNA methylation BeadChip array data
Source: Epigenetics. 2023 Sep 20;18(1):2257437. doi: 10.1080/15592294.2023.2257437 (PMC11062373; doi:10.1080/15592294.2023.2257437)
Supplement: Supplemental Material [file KEPI_A_2257437_SM7647.zip › Supplementary files/supplemental_material_editable.docx]

**Supplemental material**

**Supplementary Tables and Figures**

| Genomic Location | Odds Ratio | p Value | Input | Background |
| --- | --- | --- | --- | --- |
| 5'UTR | 0 | 1.00E+00 | 0 | 0 |
| 1stExon | 2.401 | 5.05E-27 | 0.138 | 0.062 |
| TSS1500 | 1.224 | 2.40E-03 | 0.183 | 0.154 |
| TSS200 | 2.823 | 1.32E-54 | 0.226 | 0.094 |
| 3'UTR | 0 | 1.00E+00 | 0 | 0 |
| Intergenic | 0.807 | 3.07E-04 | 0.229 | 0.269 |
| Body | 0.669 | 9.32E-15 | 0.353 | 0.449 |
| Island | 3.052 | 3.91E-93 | 0.4 | 0.18 |
| N_Shelf | 0.958 | 8.06E-01 | 0.042 | 0.044 |
| N_Shore | 0.591 | 6.82E-07 | 0.053 | 0.086 |
| OpenSea | 0.523 | 2.71E-37 | 0.403 | 0.563 |
| S_Shelf | 0.866 | 2.98E-01 | 0.039 | 0.044 |
| S_Shore | 0.75 | 4.52E-03 | 0.063 | 0.083 |

Supplementary Table 3. Enrichment analysis of genomic locations based on the 1,578 CpG sites in $S_{high}$ using the EWAS enrichment toolset. Input: The proportion of the number of probes to the total number of input probes at a certain genomic location. Background: The proportion of the number of input probes to the total number of annotated probes in 850K (EPIC) at a certain genomic location (source https://ngdc.cncb.ac.cn/ewas/toolkit).

| Trait | Odds Ratio | p Value | DMC | Background |
| --- | --- | --- | --- | --- |
| prenatal arsenic exposure | 18.97 | 3.88E-224 | 135 | 4860 |
| gestational diabetes mellitus | 9.724 | 4.01E-114 | 98 | 6599 |
| Behcet's disease | 12.277 | 4.50E-111 | 82 | 4364 |
| treated with assisted reproductive technology | 168.119 | 3.27E-86 | 25 | 118 |
| rheumatoid arthritis (RA) | 6.747 | 9.49E-46 | 49 | 4602 |
| waist circumference (WC) | 17.494 | 3.09E-36 | 21 | 766 |
| Amyloid-β plaques | 34.081 | 5.10E-30 | 13 | 249 |
| colorectal laterally spreading tumor | 3.799 | 4.64E-27 | 50 | 8245 |
| prenatal paracetamol exposure | 7.738 | 5.84E-25 | 23 | 1866 |
| neurofibrillary tangles | 52.319 | 2.44E-24 | 9 | 115 |
| child abuse | 5.781 | 3.13E-23 | 27 | 2924 |
| insulin resistance | 10.227 | 1.28E-17 | 13 | 798 |
| kidney disease | 9.052 | 2.88E-15 | 12 | 830 |
| opioid dependence (OD) | 10.94 | 3.73E-13 | 9 | 516 |
| polycystic ovary syndrome (PCOS) | 18.489 | 2.69E-08 | 4 | 137 |
| hormone therapy | 7.097 | 2.81E-07 | 6 | 526 |
| Kabuki syndrome (KS) | 3.269 | 7.76E-06 | 10 | 1891 |
| predispose for traffic-related risk behaviour | Inf | 1.06E-05 | 1 | 1 |
| panic disorder | 28.568 | 1.78E-05 | 2 | 45 |
| obesity | 1.735 | 4.36E-04 | 23 | 8134 |
| mortality | 2.025 | 8.87E-04 | 13 | 3951 |
| high saturated fatty acids diet | 2.51 | 1.01E-03 | 8 | 1965 |
| cow's milk allergy | 43.891 | 1.23E-03 | 1 | 15 |
| exercise | 2.613 | 1.40E-03 | 7 | 1652 |
| insufficient sleep | 4.651 | 2.24E-03 | 3 | 399 |
| response to dietary stilbenoids | 19.789 | 5.22E-03 | 1 | 32 |
| maternal famine exposure | 19.183 | 5.53E-03 | 1 | 33 |

Supplementary Table 4. Enrichment analysis of EWAS associations based on the 1,578 CpG sites in $S_{high}$ using the EWAS enrichment toolset. DMC: Number of probe cites that are differentially methylated in the respective trait. Background: Number of probes of the respective trait reported in the EWAS Atlas (source https://ngdc.cncb.ac.cn/ewas/toolkit).


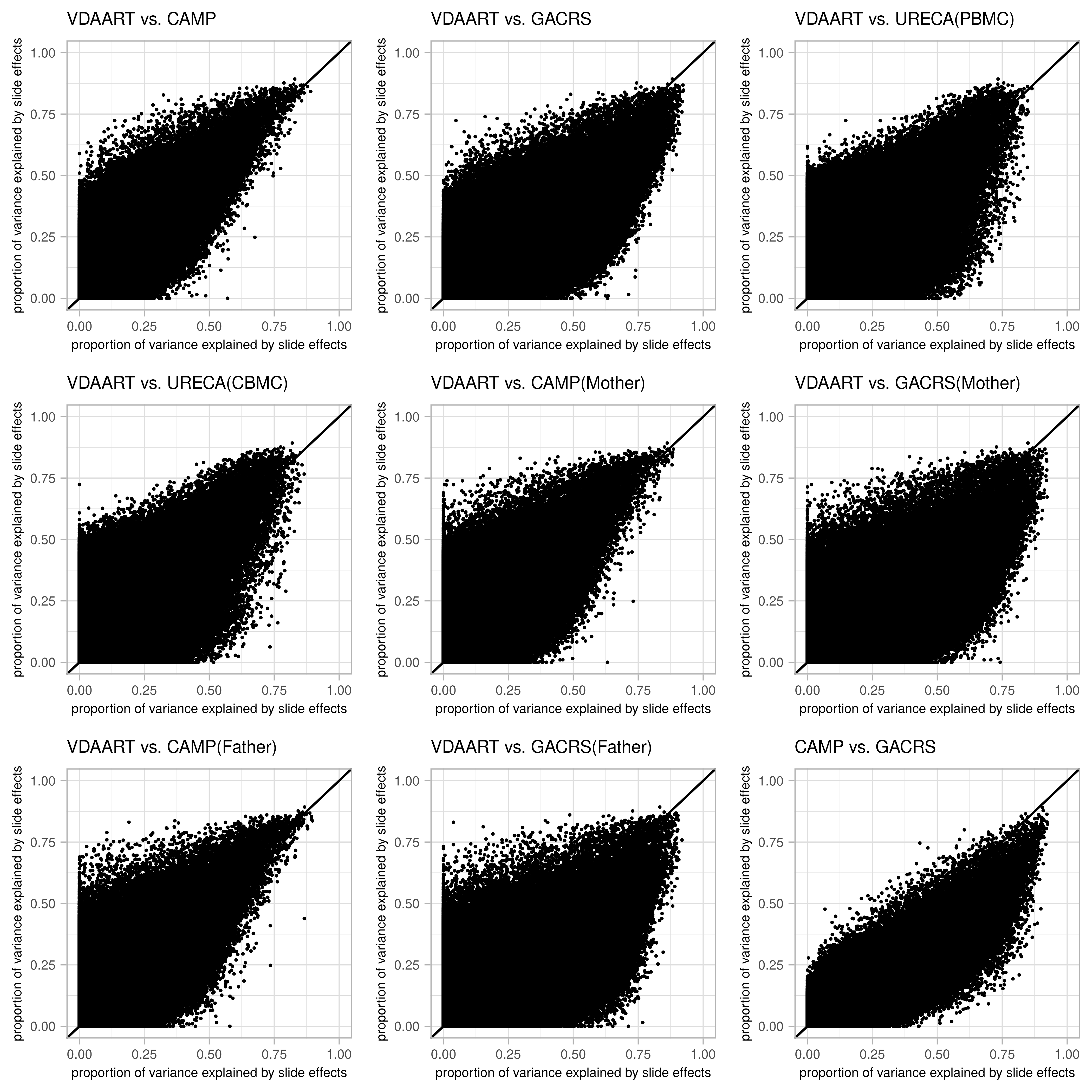


Supplementary Figure 1. Pairwise comparisons of the estimated proportion of variance explained by slide effects between the nine sub-cohorts VDAART, GACRS (offspring, mothers, and fathers), CAMP (offspring, mothers, and fathers), URECA (PBMC and CBMC) (part I).


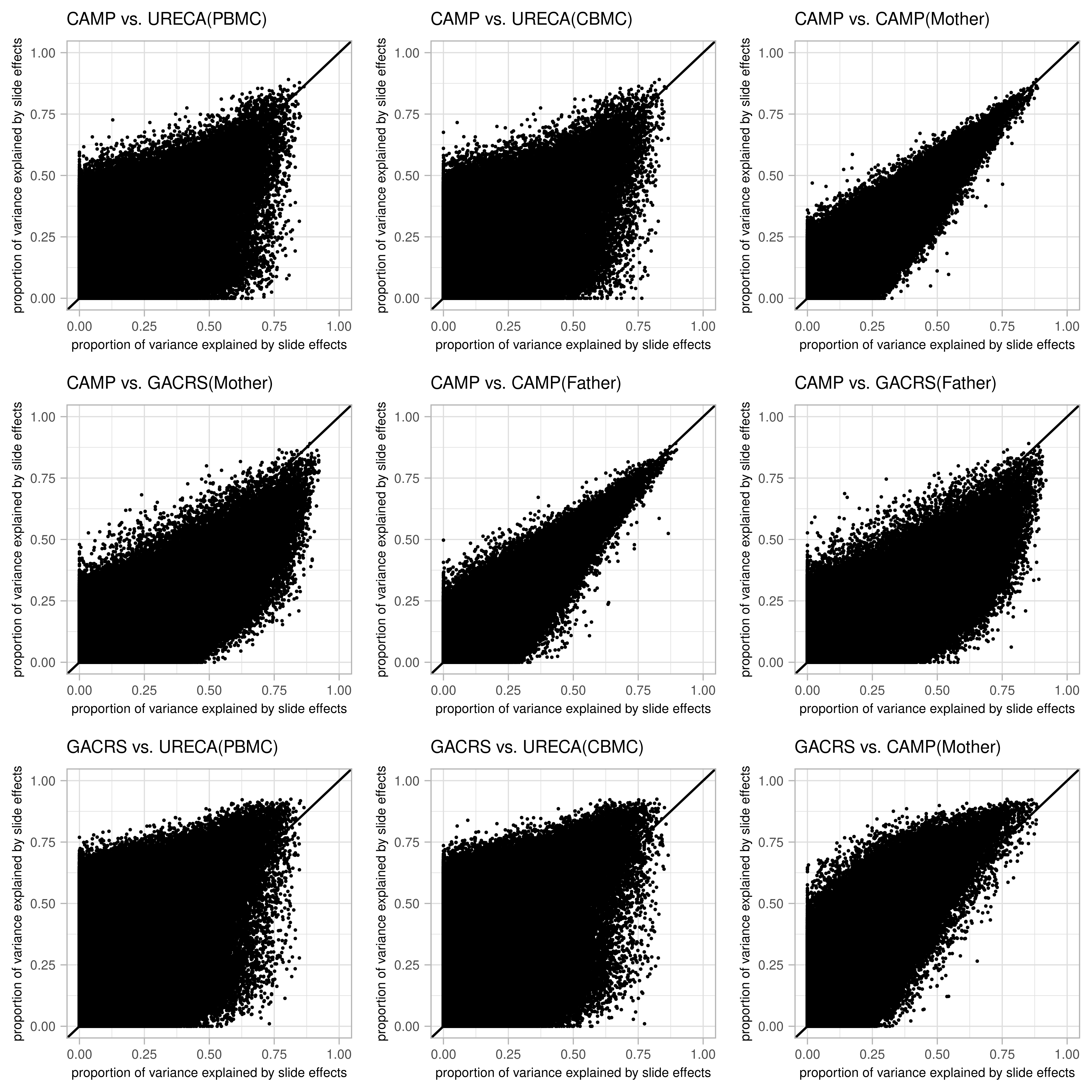


Supplementary Figure 2. Pairwise comparisons of the estimated proportion of variance explained by slide effects between the nine sub-cohorts VDAART, GACRS (offspring, mothers, and fathers), CAMP (offspring, mothers, and fathers), URECA (PBMC and CBMC) (part II).


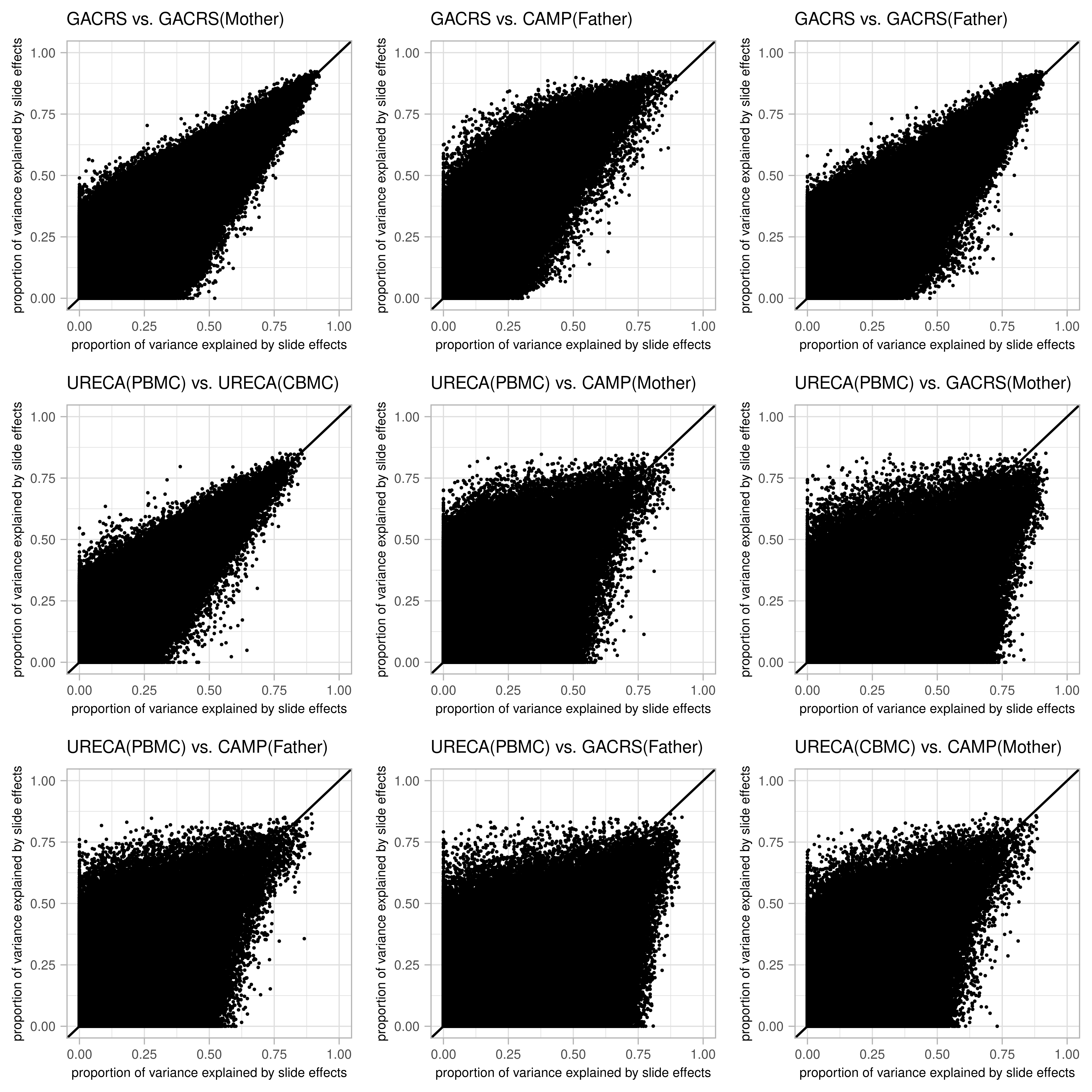


Supplementary Figure 3. Pairwise comparisons of the estimated proportion of variance explained by slide effects between the nine sub-cohorts VDAART, GACRS (offspring, mothers, and fathers), CAMP (offspring, mothers, and fathers), URECA (PBMC and CBMC) (part III).


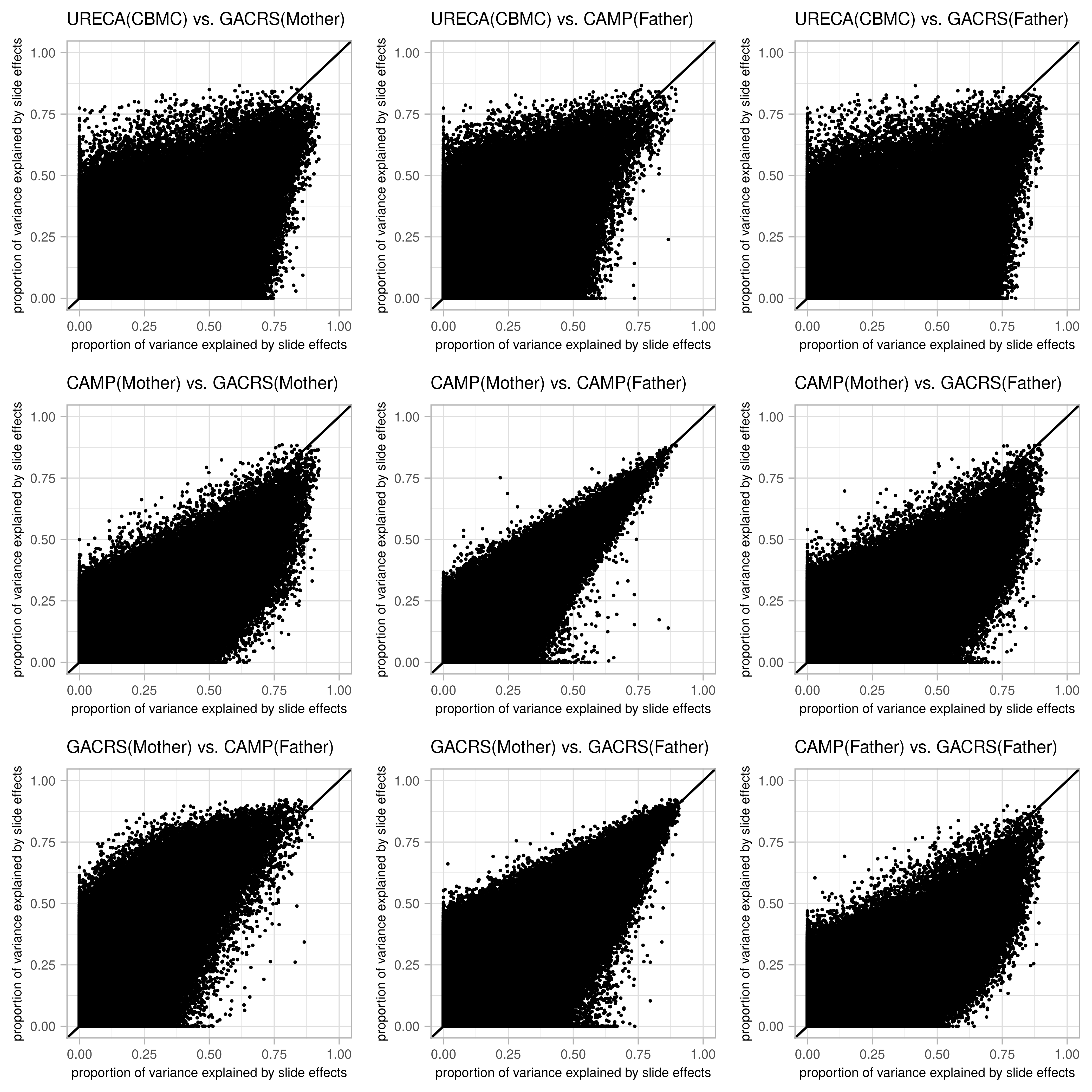


Supplementary Figure 4. Pairwise comparisons of the estimated proportion of variance explained by slide effects between the nine sub-cohorts VDAART, GACRS (offspring, mothers, and fathers), CAMP (offspring, mothers, and fathers), URECA (PBMC and CBMC) (part IV).


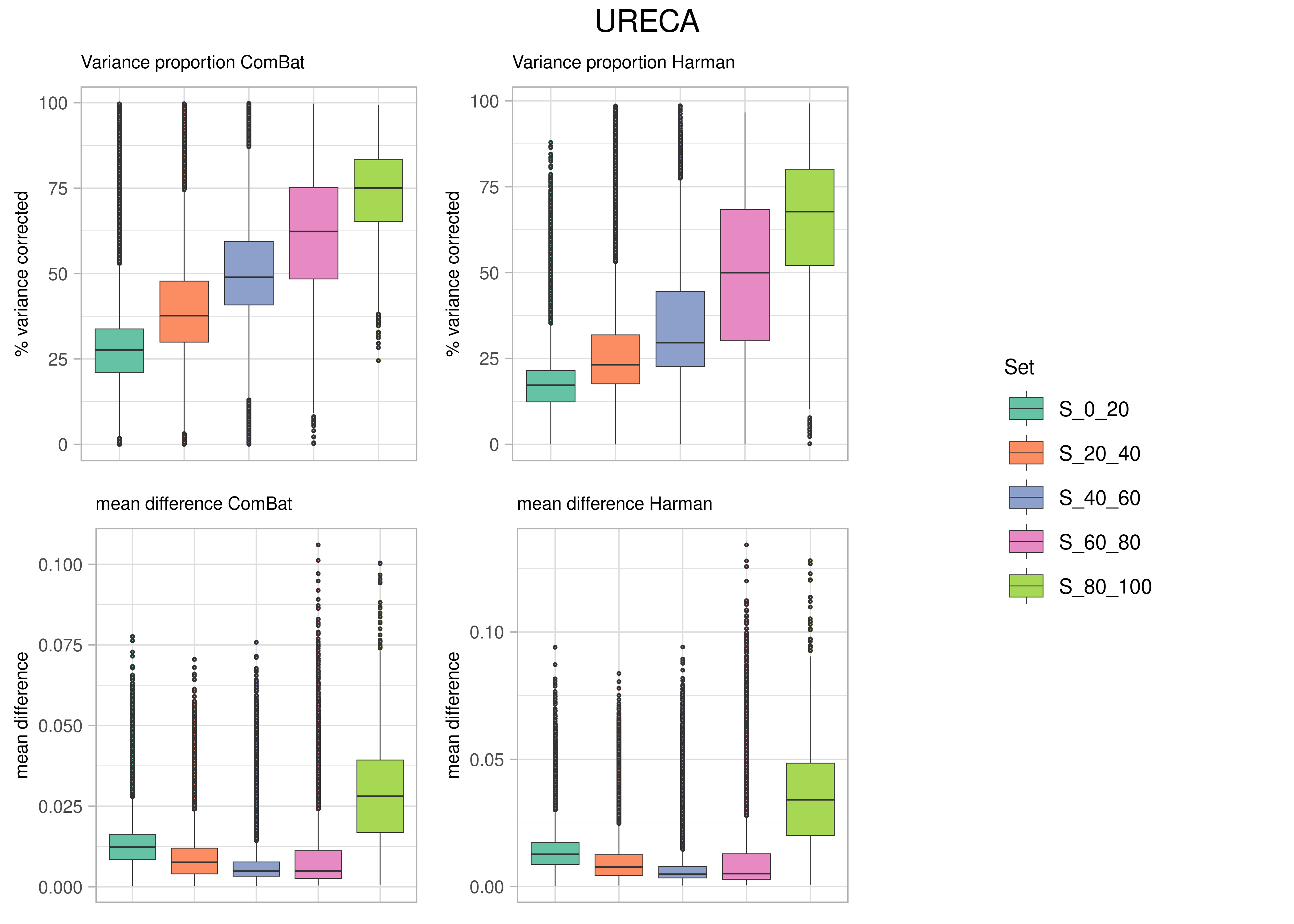


Supplementary Figure 5. Results by Ross et al.^1^ for the URECA dataset, partitioned according to the sets $S_{0-20}, S_{20-40}, S_{40-60}, S_{60-80,}$ and $S_{80-100}$.


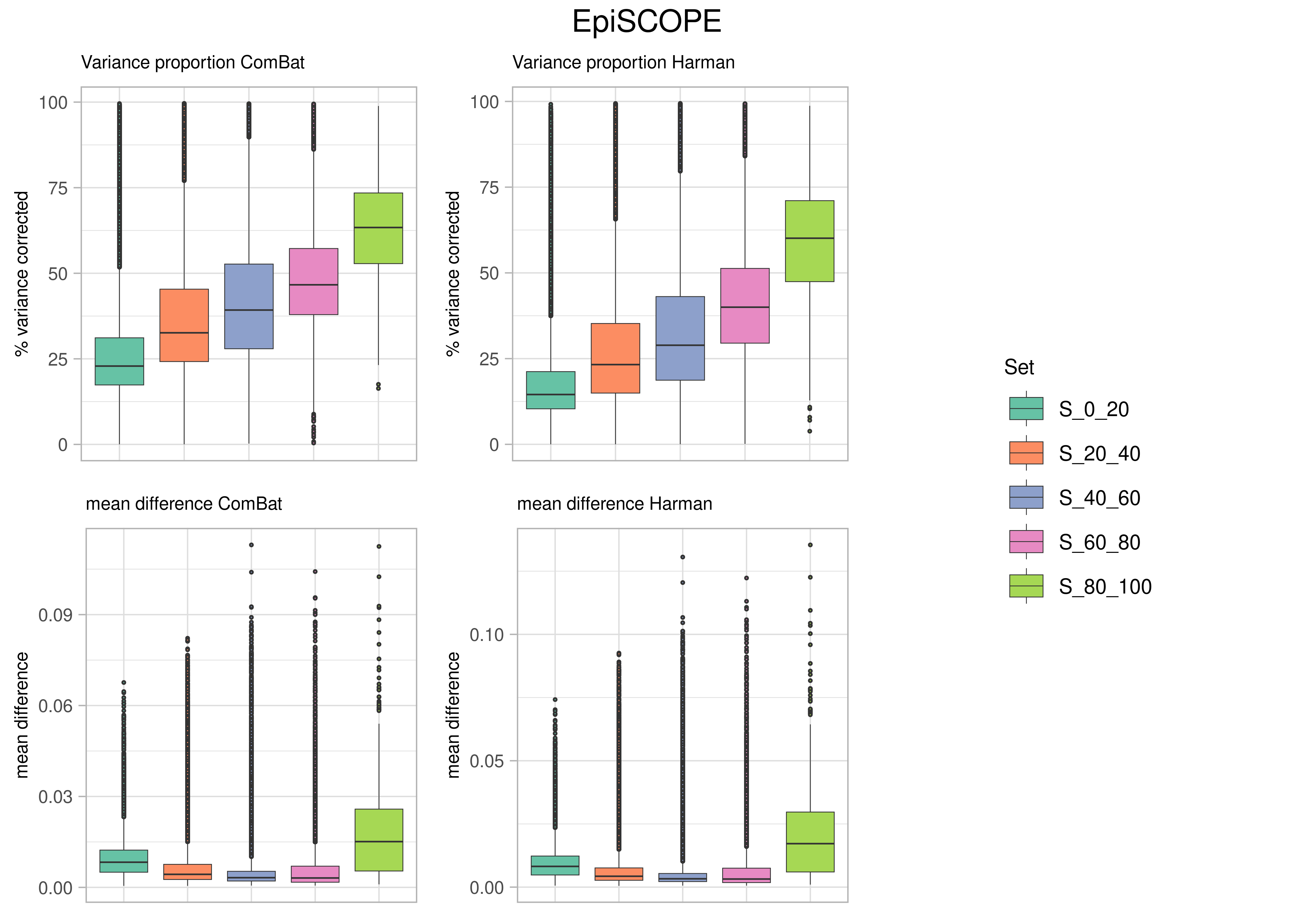


Supplementary Figure 6. Results by Ross et al.^1^ for the EpiSCOPE dataset, partitioned according to the sets $S_{0-20}, S_{20-40}, S_{40-60}, S_{60-80,}$and $S_{80-100}$.


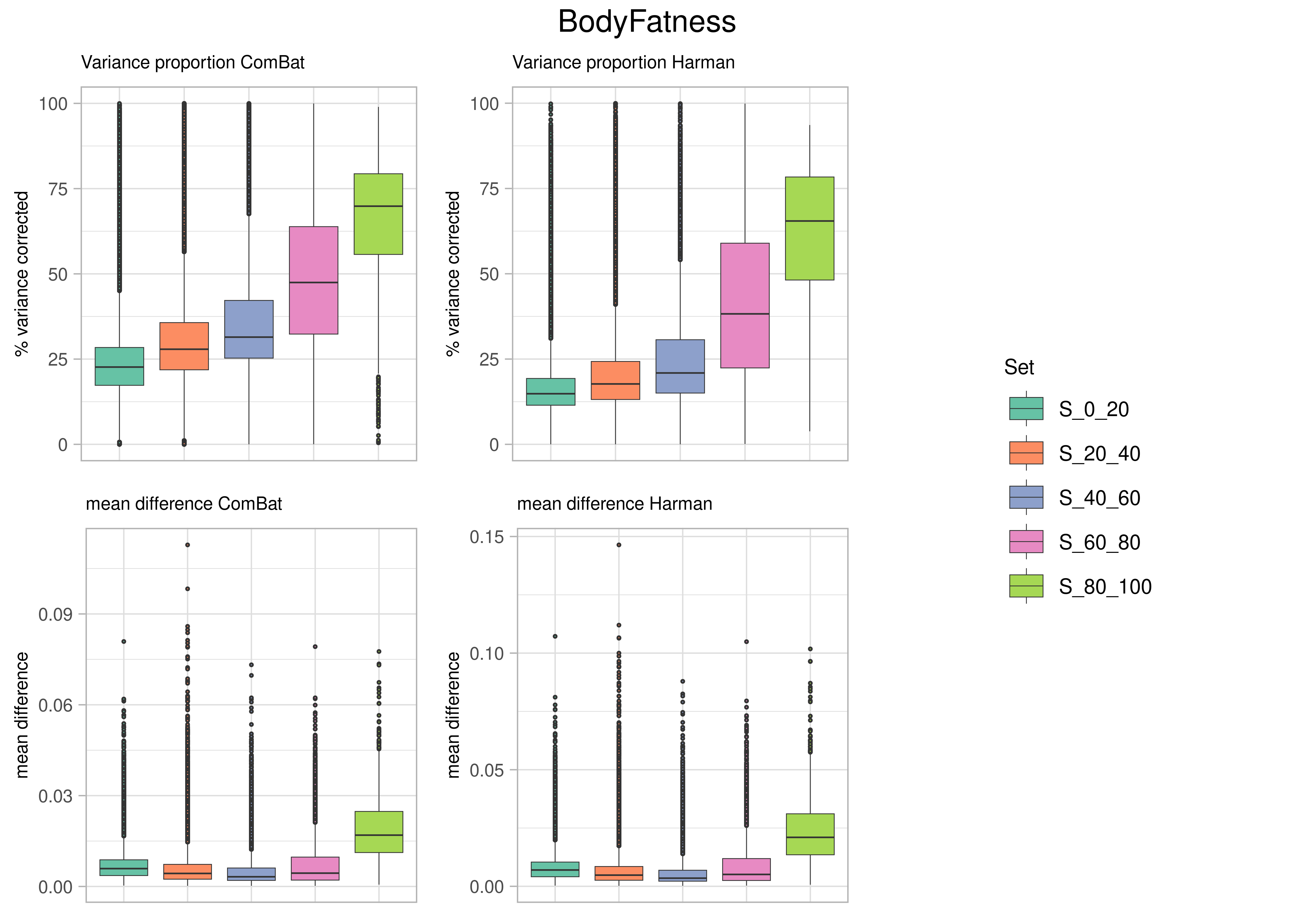


Supplementary Figure 7. Results by Ross et al.^1^ for the BodyFatness dataset, partitioned according to the sets $S_{0-20}, S_{20-40}, S_{40-60}, S_{60-80,}$and $S_{80-100}$.


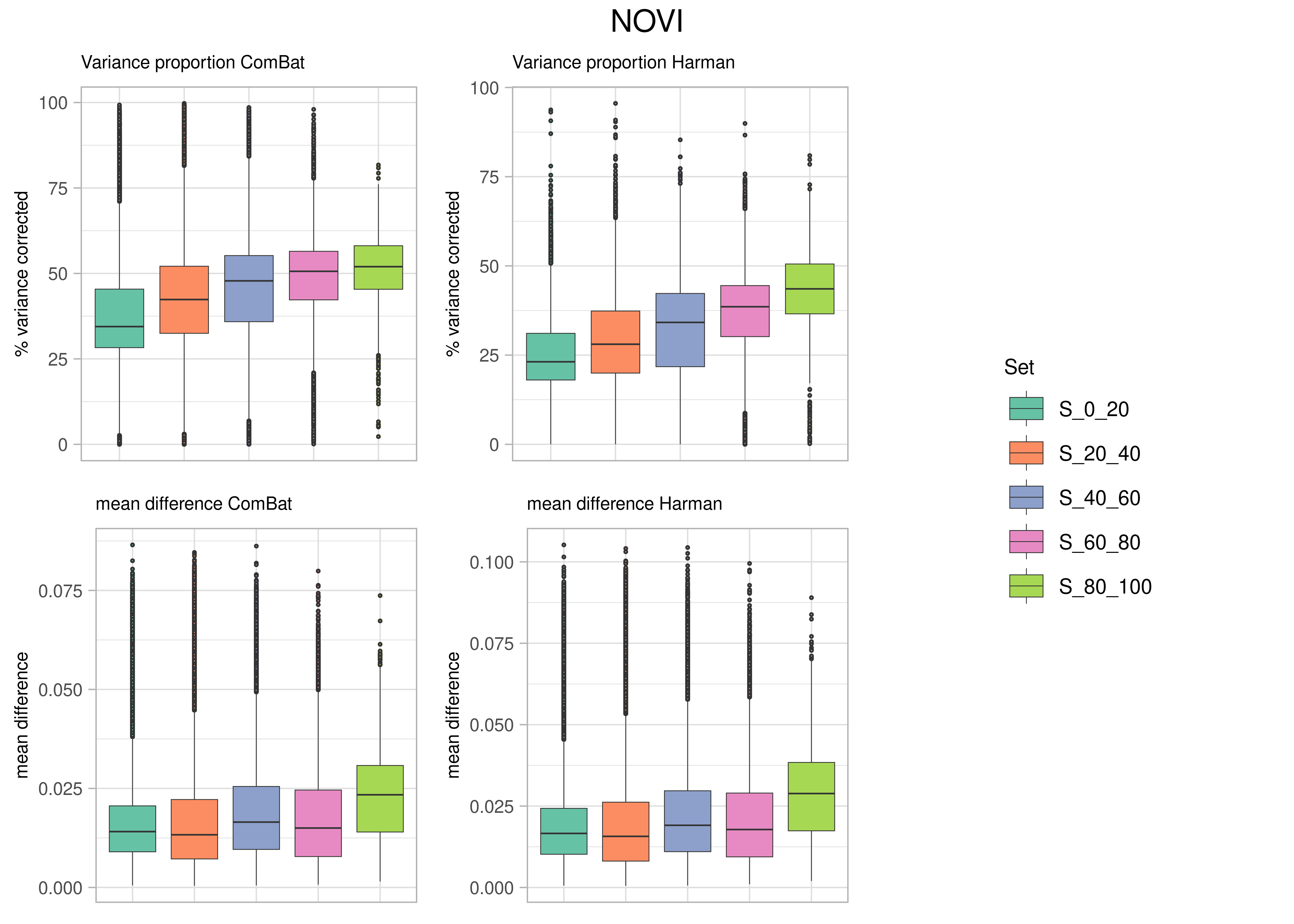


Supplementary Figure 8. Results by Ross et al.^1^ for the NOVI dataset, partitioned according to the sets $S_{0-20}, S_{20-40}, S_{40-60}, S_{60-80,}$and $S_{80-100}$.


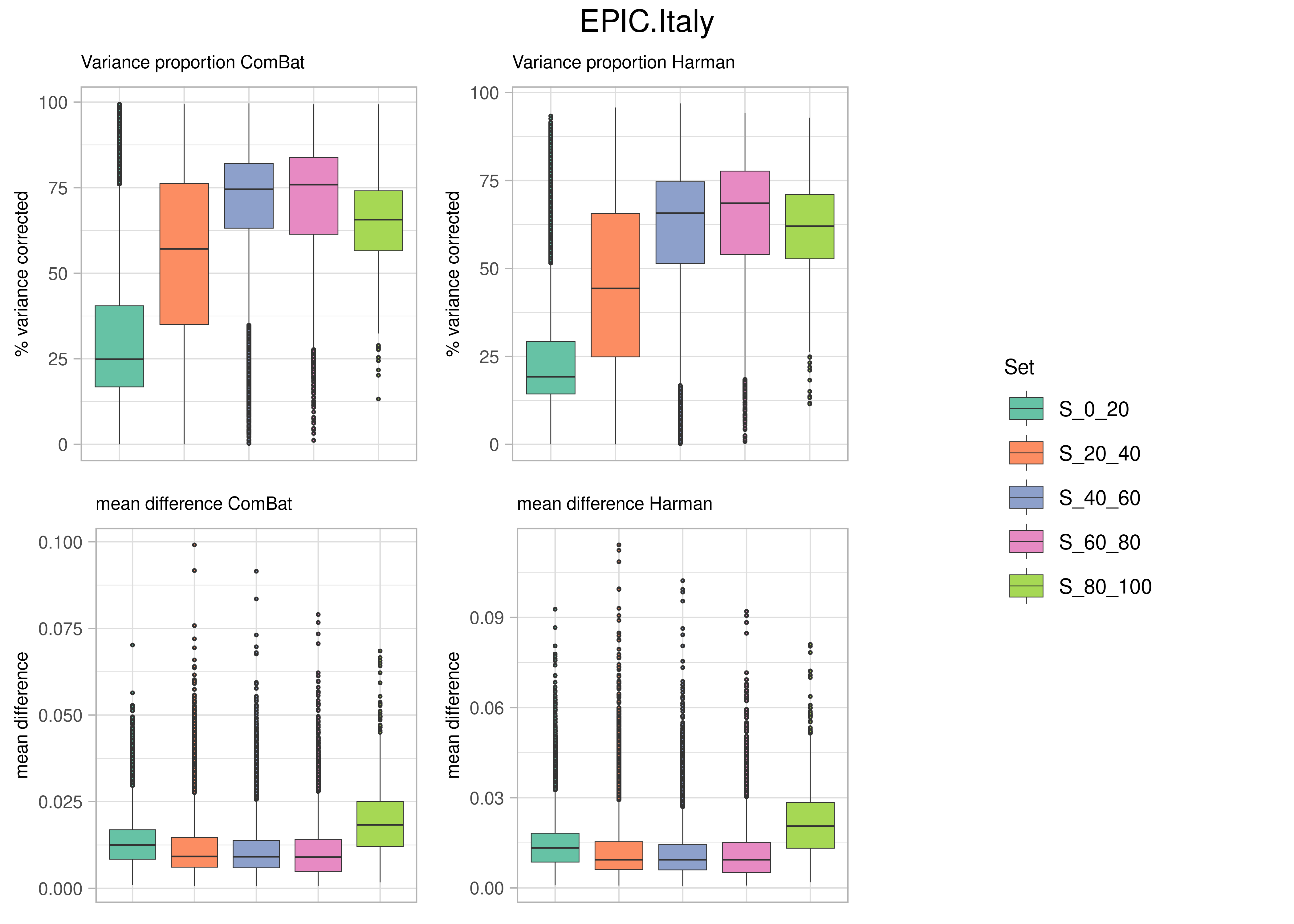


Supplementary Figure 9. Results by Ross et al.^1^ for the EPIC.Italy dataset, partitioned according to the sets $S_{0-20}, S_{20-40}, S_{40-60}, S_{60-80,}$and $S_{80-100}$.


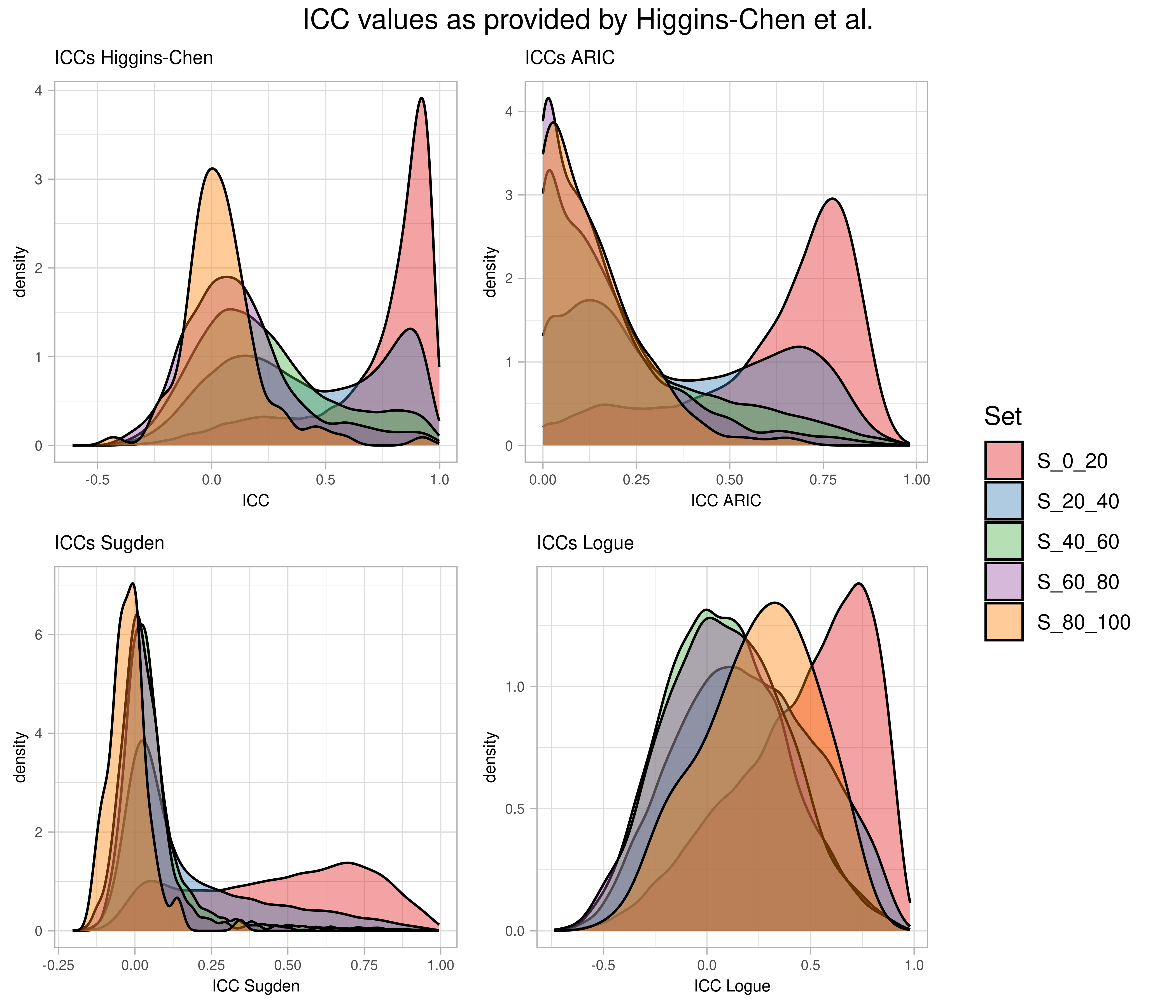


Supplementary Figure 10. Density plots of interclass correlation coeffcients of DNA methylation measurements based on technical replicates as provided by Higgins-Chen et al.^2^, stratified by the sets $S_{0-20}, S_{20-40}, S_{40-60}, S_{60-80,}$and $S_{80-100}$.


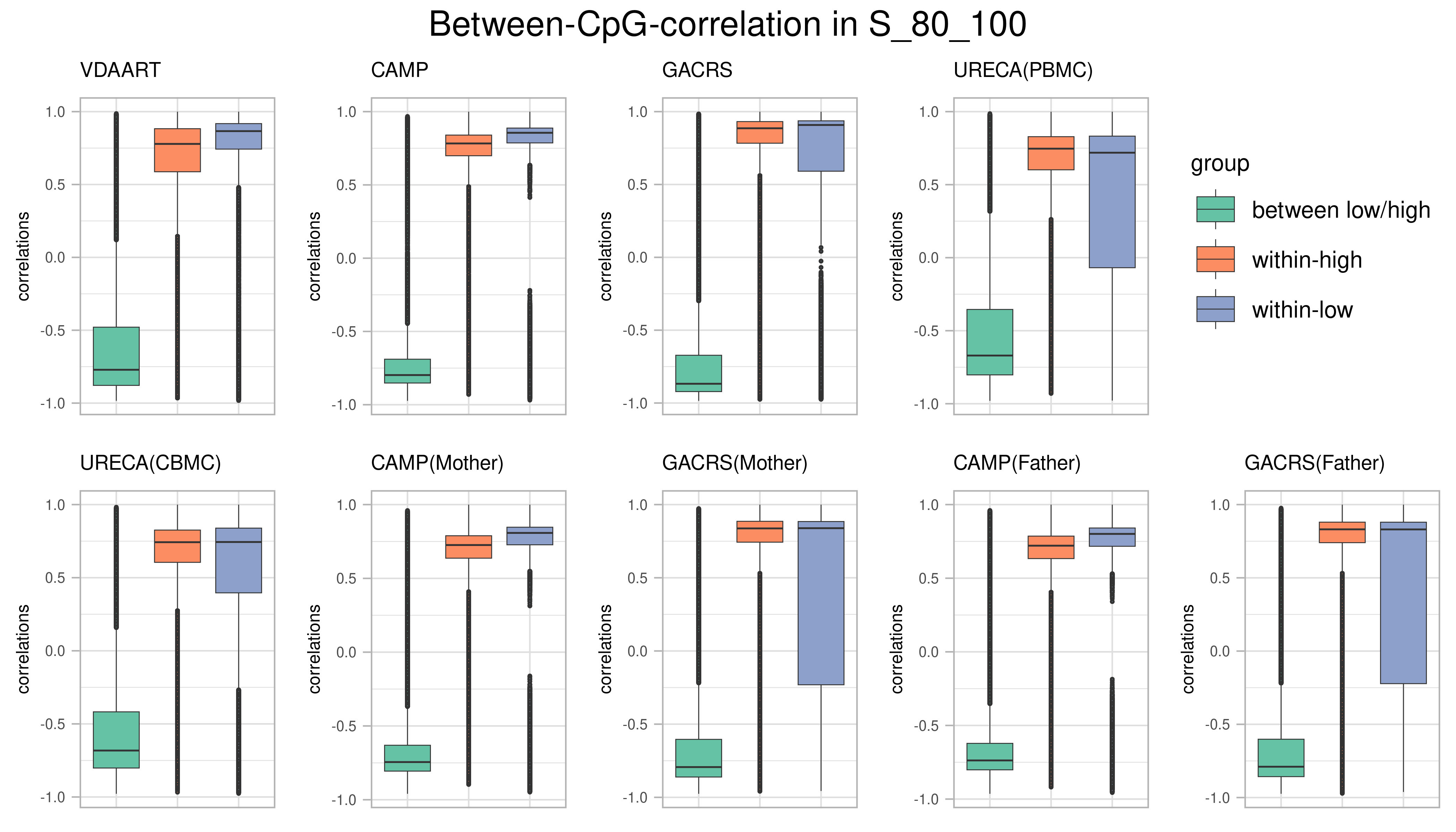


Supplementary Figure 11. Between-CpG-correlations between CpG sites in $S_{80\_100}$, partitioned by median covariate-adjusted methylation beta-values (50% cutoff) in the respective cohort.


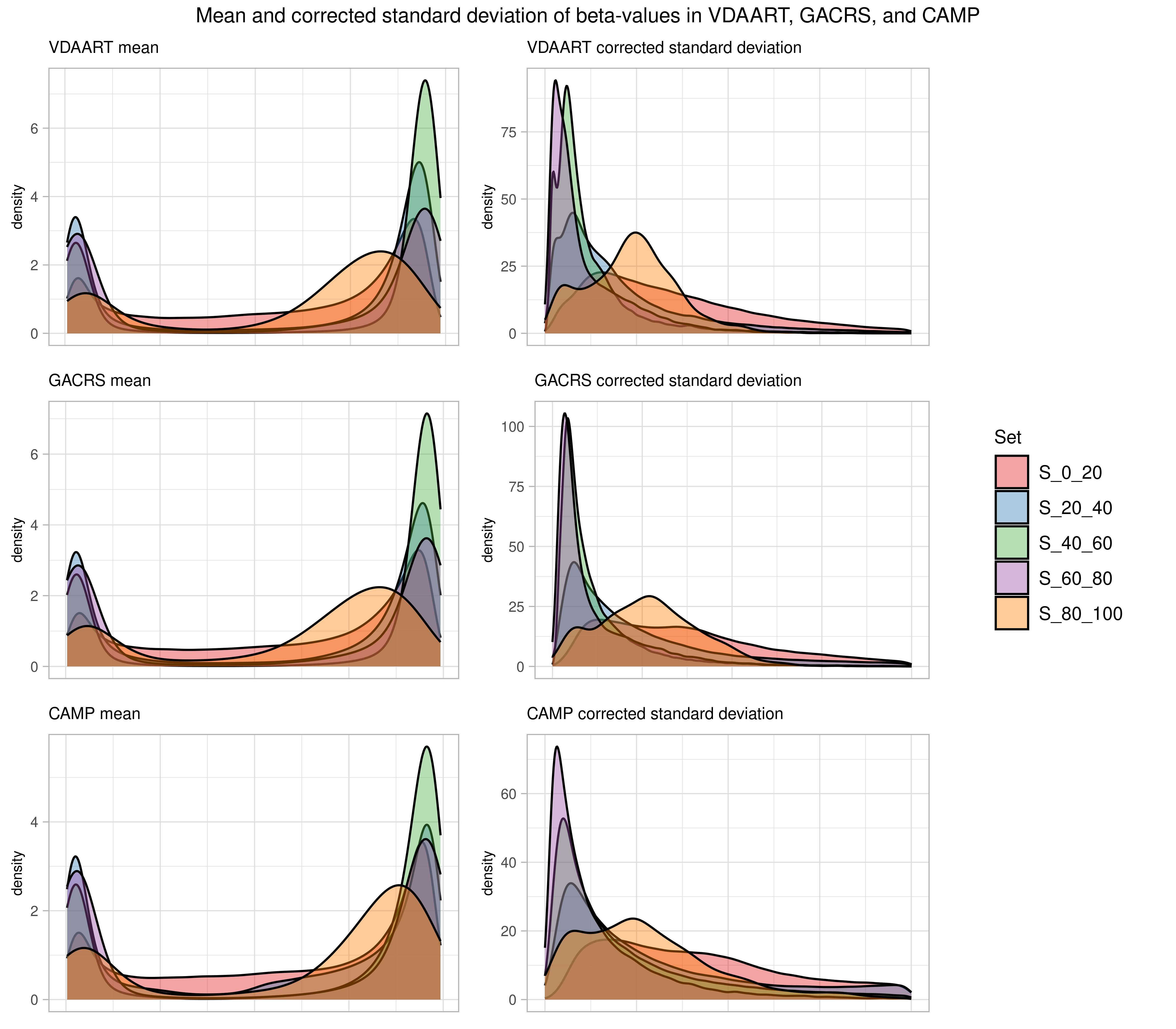


Supplementary Figure 12. Density plots of mean and corrected standard deviation of methylation beta-values in VDAART, GACRS offspring, and CAMP offspring, stratified by the sets $S_{0-20}, S_{20-40}, S_{40-60}, S_{60-80,}$and $S_{80-100}$. The corrected standard deviation adjusts for the variance explained by slide effects (estimated).


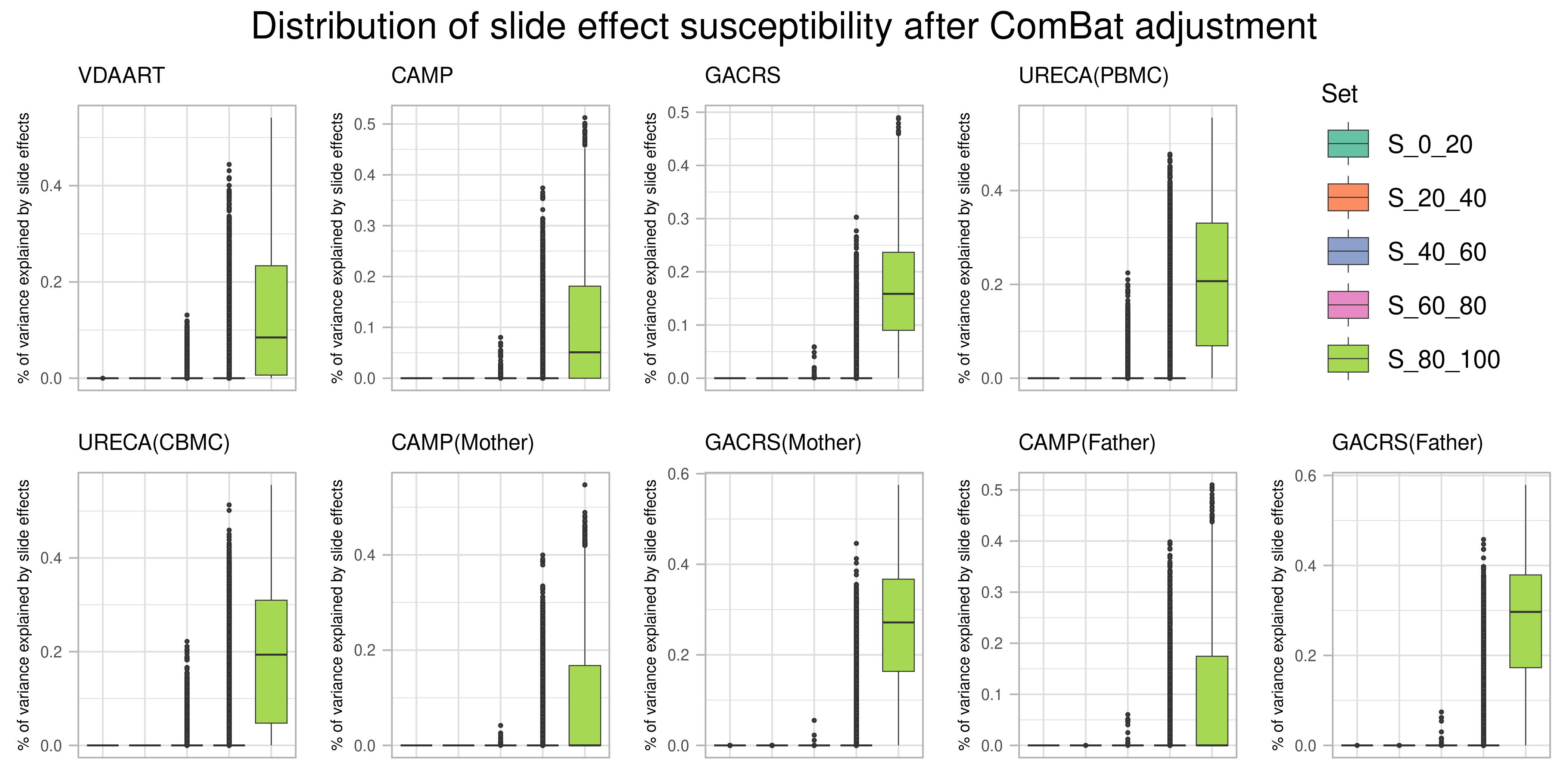


Supplementary Figure 13. Proportions of variance explained by slide effects in VDAART, CAMP (offspring, mothers, fathers), GACRS (offspring, mothers, fathers), and URECA (PBMC and CBMC), partitioned by $S_{0-20}, S_{20-40}, S_{40-60}, S_{60-80,}$and $S_{80-100}$, after slide correction with ComBat (Adjustment Method II).


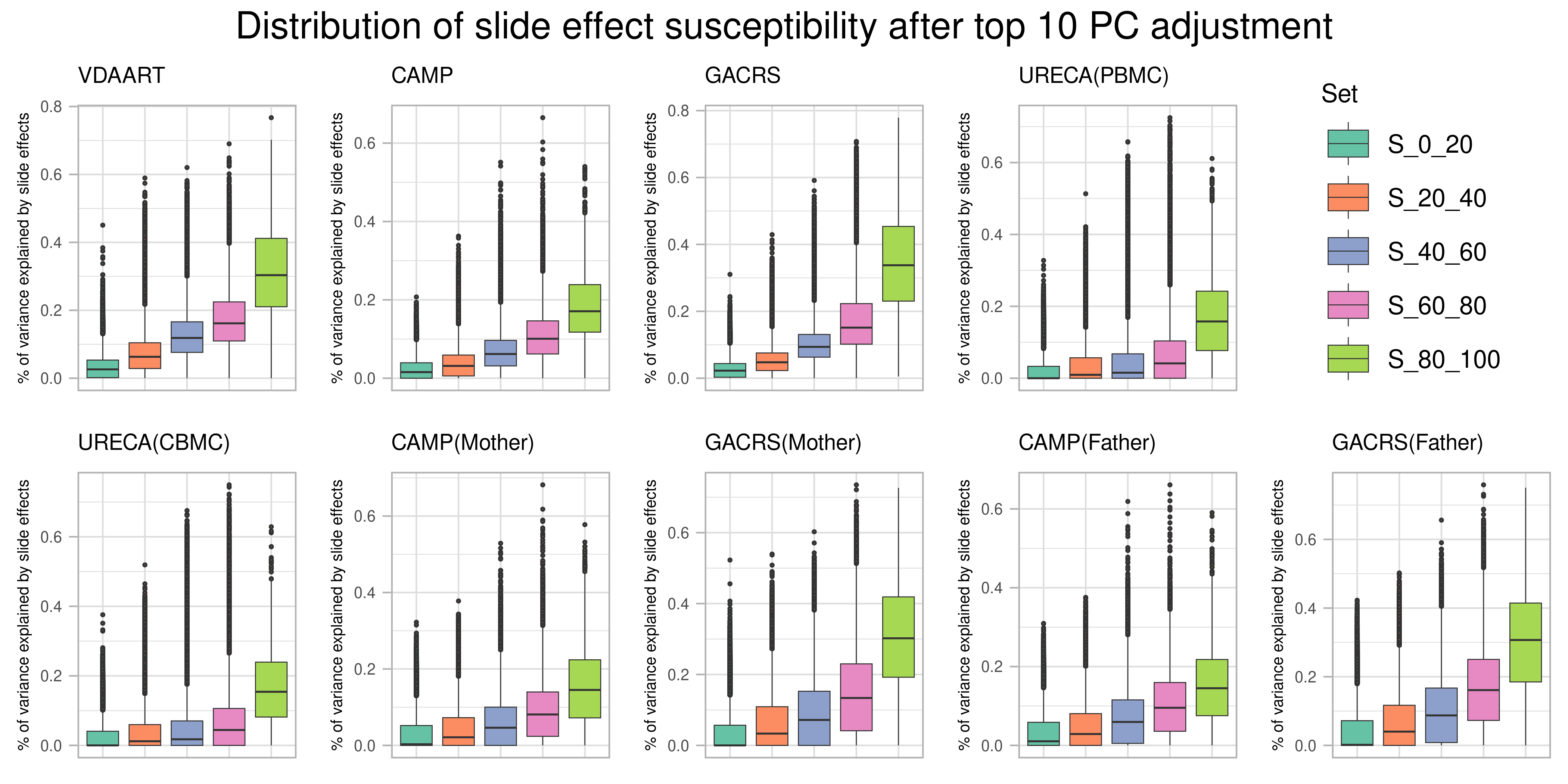


Supplementary Figure 14. Proportions of variance explained by slide effects in VDAART, CAMP (offspring, mothers, fathers), GACRS (offspring, mothers, fathers), and URECA (PBMC and CBMC), partitioned by $S_{0-20}, S_{20-40}, S_{40-60}, S_{60-80,}$and $S_{80-100}$, after slide correction with the top 10 PCs based on methylome-wide data (Adjustment Method III).


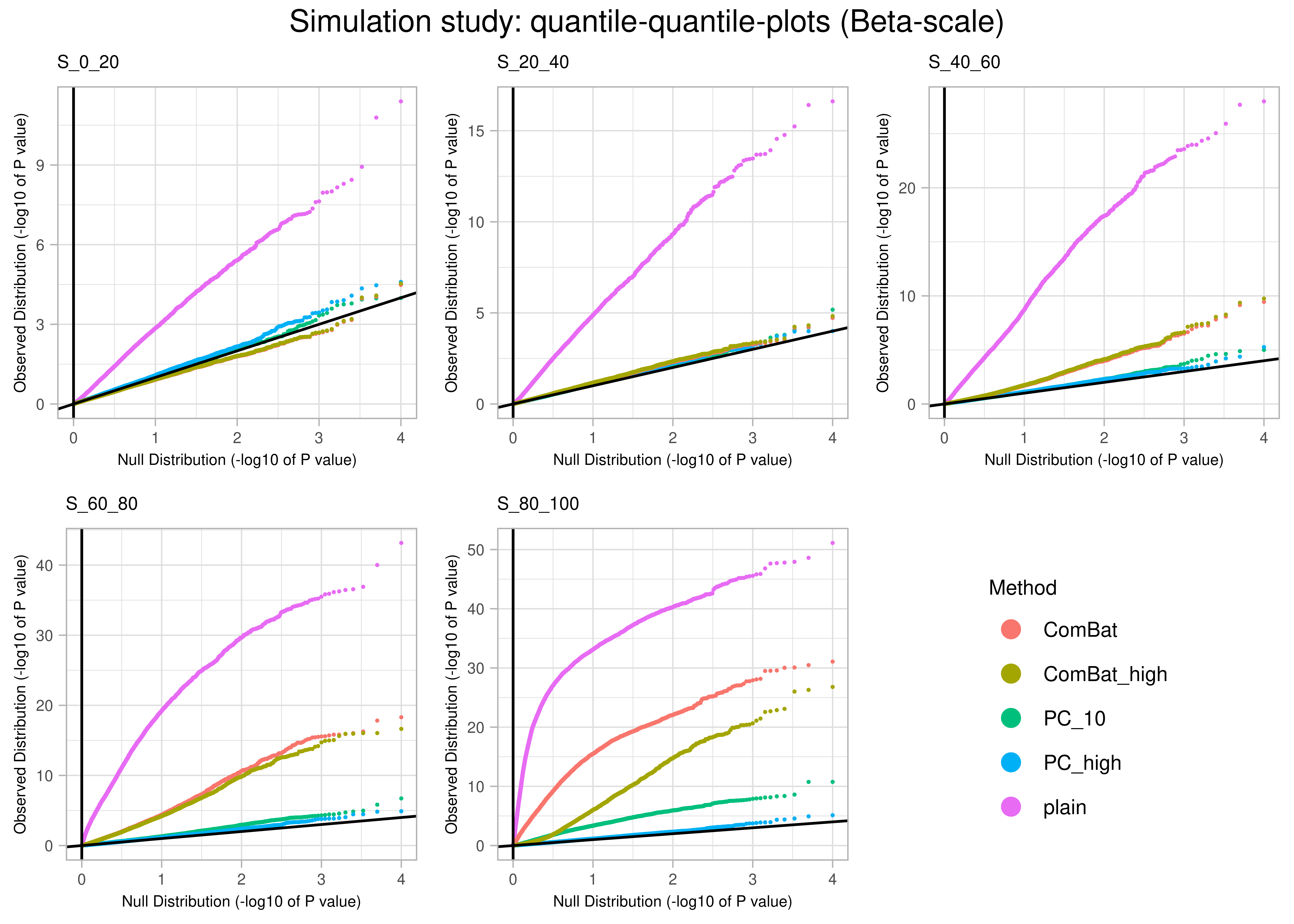


Supplementary Figure 15. Quantile-quantile-plots (qq-plots) for the simulation study based on VDAART data. The qq-plots are displayed for the five sets $S_{0-20}, S_{20-40}, S_{40-60}, S_{60-80,}$and $S_{80-100}$ separately. Five different analysis approaches are included: plain (no correction, linear regression), PC_10 (first ten principal components based on all CpGs on $C_{odd}$ as covariates in linear regression), PC_high (first principal component based on CpGs on $C_{odd}$ in $S_{high}$ as a covariate in linear regression), ComBat (ComBat adjustment based on CpGs on $C_{odd}$on M-scale, linear regression), and ComBat_high (ComBat adjustment based on all CpGs on $C_{odd}$performed in $S_{high}$ and all other CpGs separately on M-scale, linear regression). Methylation values were considered on the beta-scale. Association analysis and adjustment were solely based on $C_{odd}$ data, phenotype simulation based on$C_{even}$ data only. $C_{odd}$: chromosomes with odd numbers; $C_{even}$: chromosomes with even numbers.


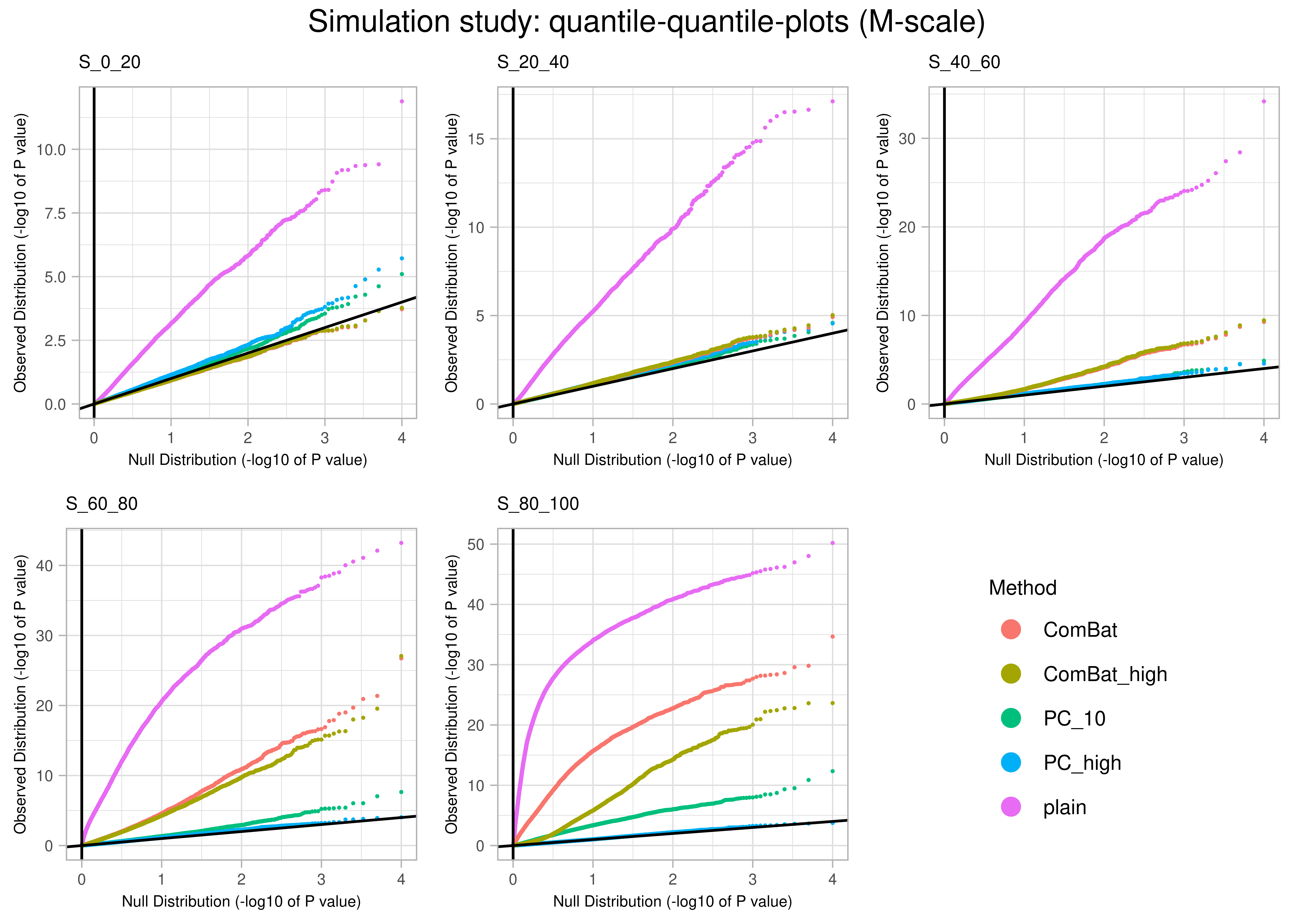


Supplementary Figure 16. Quantile-quantile-plots (qq-plots) for the simulation study based on VDAART data. The qq-plots are displayed for the five sets $S_{0-20}, S_{20-40}, S_{40-60}, S_{60-80,}$and $S_{80-100}$ separately. Five different analysis approaches are included: plain (no correction, linear regression), PC_10 (first ten principal components based on all CpGs on $C_{odd}$ as covariates in linear regression), PC_high (first principal component based on CpGs on $C_{odd}$ in $S_{high}$ as a covariate in linear regression), ComBat (ComBat adjustment based on CpGs on $C_{odd}$on M-scale, linear regression), and ComBat_high (ComBat adjustment based on all CpGs on $C_{odd}$performed in $S_{high}$ and all other CpGs separately on M-scale, linear regression). Methylation values were considered on the M-scale. Association analysis and adjustment were solely based on $C_{odd}$ data, phenotype simulation based on$C_{even}$ data only. $C_{odd}$: chromosomes with odd numbers; $C_{even}$: chromosomes with even numbers.

**Supplementary Methods**

**Study populations**

The Genetic Epidemiology of Asthma in Costa Rica Study (GACRS): GACRS represents a Hispanic population isolate from the Central Valley of Costa Rica with one of the highest prevalence of asthma worldwide (23% in children)^3^. A total of 1,165 children aged 6-14 years with mild to moderate asthma were enrolled. GACRS and CAMP (see below) used identical protocols for phenotypes and asthma diagnoses.

Childhood Asthma Management Program (CAMP): CAMP is a multicenter, randomized, double-masked clinical trial designed to determine the long-term effects of inhaled treatments for mild to moderate childhood asthma^4^. The study includes 1,041 asthmatic children (32% from ethnic minority groups), aged 5 to 12 years at screening.

Vitamin D Antenatal Asthma Reduction Trial (VDAART): VDAART is a randomized, double-blind, placebo-controlled study of vitamin D vs. placebo. Children had whole blood samples obtained at the ages of 1, 3, and 6, as well as cord blood samples at delivery. DNA methylation for the described analyses is based on cord blood.

Urban Environment and Childhood Asthma (URECA)

URECA is a birth cohort study initiated in 2005 in Baltimore, Boston, New York City and St. Louis. Pregnant women were recruited and either they or the father of their unborn child had a history of asthma, allergic rhinitis, or eczema^5^.

**Preprocessing and normalization for GACRS, CAMP and VDAART**

DNA methylation data for GACRS and CAMP was generated as part of the NHLBI Trans-Omics for Precision Medicine (TOPMed) program using the standard protocol for the Illumina Infinium MethylationEPIC BeadChip array (Illumina, USA), as implemented at the Keck Molecular Genomics Core Facility. DNA methylation data for VDAART was generated using the standard protocol for the Illumina Infinium MethylationEPIC BeadChip array, as implemented at the Channing lab.

The probe-level and sample-level cleaning for GACRS, CAMP, and VDAART was performed using the same following pipeline. Data preprocessing and quality control was performed using minfi^6^. We used meffil^7^ for sample level outlier filtering. As part of the CpG site-based filtering, we removed probes with low detection pvalues (cut off 0.05), non-CpG probes (CH probes), probes containing SNPs at the CpG interrogation or at the single nucleotide extension and sex chromosomes. We calculated the blood cell count estimates using the Houseman algorithm^8,9^ using whole blood cell types as reference for GACRS and CAMP and optimized algorithm in Salas et al.^10,11^, for cord blood cell type deconvolution for VDAART. Background correction and data normalization was performed using functional normalization^12^ followed by type II probe and technical bias correction using Regression on Correlated probes (RCP) method^13^.

**Preprocessing for URECA**

A detailed description of the DNA methylation data processing for the URECA data is described in the corresponding publication^5^. We downloaded the beta-value matrix and the phenotype information that is available from <https://www.ncbi.nlm.nih.gov/geo/query/acc.cgi?acc=GSE132181>. Based on this data, we extracted beta-values, sex, slide information, and the first two ancestral principal components for 194 CBMC and 195 PBMC samples. Based on the corresponding beta-values, we estimated cell type proportions using the epidish function in the *ENmix* R package (reference centDHSbloodDMC, method “RPC”) for further adjustment.

**Adjustment approaches**

Adjustment Method I:

1. Beta-values to M-values transformation
2. Regression M-value ~ covariates
3. Residual M-values to Beta-values transformation (intercept maintained in residuals)

Adjustment Method II:

1. Beta-values to M-values transformation
2. Combat slide effect adjustment while also adjusting for covariates
3. Residual M-values to Beta-values transformation (intercept maintained in residuals)

Adjustment Method III:

1. Beta-values to M-values transformation
2. PCA analysis of M-values
3. Regression M-value ~ covariates + 10 PCs
4. Residual M-values to Beta-values transformation (intercept maintained in residuals)

Covariates:

VDAART: sex, cell type proportions, race

GACRS offspring: sex, age, cell type proportions

GACRS mothers: age, cell type proportions

GACRS fathers: age, cell type proportions

CAMP offspring: sex, age, cell type proportions, race

CAMP mothers: age, cell type proportions

CAMP fathers: age, cell type proportions

URECA PBMC: sex, cell type proportions, first two ancestral principal components

URECA CBMC: sex, cell type proportions, first two ancestral principal components

Adjustment for estimated cell type proportions for all cohorts was based on the first two principal components of the corresponding estimated cell type proportion matrix.

**References**

1. Ross, J. P. *et al.* Batch-effect detection, correction and characterisation in Illumina HumanMethylation450 and MethylationEPIC BeadChip array data. *Clin. Epigenetics* **14**, 58 (2022).

2. Higgins-Chen, A. T. *et al.* A computational solution for bolstering reliability of epigenetic clocks: Implications for clinical trials and longitudinal tracking. *Nat. Aging* **2**, 644–661 (2022).

3. Soto-Quiros, M. *et al.* The prevalence of childhood asthma in Costa Rica. *Clin. Exp. Allergy J. Br. Soc. Allergy Clin. Immunol.* **24**, 1130–1136 (1994).

4. The Childhood Asthma Management Program (CAMP): Design, Rationale, and Methods. *Control. Clin. Trials* **20**, 91–120 (1999).

5. McKennan, C. *et al.* Longitudinal data reveal strong genetic and weak non-genetic components of ethnicity-dependent blood DNA methylation levels. *Epigenetics* **16**, 662–676 (2021).

6. Aryee, M. J. *et al.* Minfi: a flexible and comprehensive Bioconductor package for the analysis of Infinium DNA methylation microarrays. *Bioinforma. Oxf. Engl.* **30**, 1363–1369 (2014).

7. Min, J. L., Hemani, G., Davey Smith, G., Relton, C. & Suderman, M. Meffil: efficient normalization and analysis of very large DNA methylation datasets. *Bioinforma. Oxf. Engl.* **34**, 3983–3989 (2018).

8. Houseman, E. A. *et al.* DNA methylation arrays as surrogate measures of cell mixture distribution. *BMC Bioinformatics* **13**, 86 (2012).

9. Houseman, E. A. *et al.* Reference-free deconvolution of DNA methylation data and mediation by cell composition effects. *BMC Bioinformatics* **17**, 259 (2016).

10. Salas, L. A. *et al.* An optimized library for reference-based deconvolution of whole-blood biospecimens assayed using the Illumina HumanMethylationEPIC BeadArray. *Genome Biol.* **19**, 64 (2018).

11. Gervin, K. *et al.* Systematic evaluation and validation of reference and library selection methods for deconvolution of cord blood DNA methylation data. *Clin. Epigenetics* **11**, 125 (2019).

12. Fortin, J.-P. *et al.* Functional normalization of 450k methylation array data improves replication in large cancer studies. *Genome Biol.* **15**, 503 (2014).

13. Niu, L., Xu, Z. & Taylor, J. A. RCP: a novel probe design bias correction method for Illumina Methylation BeadChip. *Bioinforma. Oxf. Engl.* **32**, 2659–2663 (2016).
